# A CMOS–memristor hybrid system for implementing stochastic binary spike timing-dependent plasticity

**DOI:** 10.1098/rsta.2021.0018

**Published:** 2022-07-25

**Authors:** Javad Ahmadi-Farsani, Saverio Ricci, Shahin Hashemkhani, Daniele Ielmini, Bernabé Linares-Barranco, Teresa Serrano-Gotarredona

**Affiliations:** ^1^ Instituto de Microelectrónica de Sevilla, IMSE-CNM (CSIC and Universidad de Sevilla), Av. Américo Vespucio 28, 41092 Sevilla, Spain; ^2^ Dipartimento di Elettronica, Informazione e Bioingegneria, Politecnico di Milano, Piazza L. da Vinci 32, 20133 Milano, Italy

**Keywords:** spike timing-dependent plasticity, stochastic-binary STDP, non-volatile memristors, spiking neural networks, CMOS analogue neurons, analogue current scaling

## Abstract

This paper describes a fully experimental hybrid system in which a 4×4 memristive crossbar spiking neural network (SNN) was assembled using custom high-resistance state memristors with analogue CMOS neurons fabricated in 180 nm CMOS technology. The custom memristors used NMOS selector transistors, made available on a second 180 nm CMOS chip. One drawback is that memristors operate with currents in the micro-amperes range, while analogue CMOS neurons may need to operate with currents in the pico-amperes range. One possible solution was to use a compact circuit to scale the memristor-domain currents down to the analogue CMOS neuron domain currents by at least 5–6 orders of magnitude. Here, we proposed using an on-chip compact current splitter circuit based on MOS ladders to aggressively attenuate the currents by over 5 orders of magnitude. This circuit was added before each neuron. This paper describes the proper experimental operation of an SNN circuit using a 4×4 1T1R synaptic crossbar together with four post-synaptic CMOS circuits, each with a 5-decade current attenuator and an integrate-and-fire neuron. It also demonstrates one-shot winner-takes-all training and stochastic binary spike-timing-dependent-plasticity learning using this small system.

This article is part of the theme issue ‘Advanced neurotechnologies: translating innovation for health and well-being’.

## 1. Introduction

In recent years, neuromorphic engineering has attracted great interest in both academia and industry because of its potential for providing energy-efficient artificial cognitive sensory and processing systems that imitate brain functions. Neuromorphic computing and engineering is highly multidisciplinary. It encompasses high-level computational neuroscience for unravelling the computing and learning principles used in biological brains, novel hardware-friendly parallel architectures capable of mapping brain computing principles on fast, efficient hardware platforms, novel circuits that imitate event-driven brain computations, and new nanoscale devices that can be used directly as synaptic or neuron primitives. One key difference between classic computers and neuromorphic computing hardware is the latter’s circumvention of the Von Neumann bottleneck [1]. In classic computers, processing elements and memory storage are physically separated and a great amount of energy is consumed in massive data transfers between processors and the different hierarchical levels of memory. In neuromorphic hardware, however, it is possible to co-locate knowledge (stored dynamically as synaptic weights) and information processing (typically performed jointly by synapses and neurons), thus reducing such continuous massive data transfers. One way of co-locating memory and processing is to use memristor devices, which can be fabricated monolithically on top of CMOS neurons [2]. In this regard, many researchers have proposed building hybrid CMOS–memristor neuromorphic computing systems. However, one issue that arises when trying to interconnect memristor-based synapses with compact CMOS neurons is the big difference between their operating currents. Memristor devices typically have an ON-resistance in the range of 2–20 kΩ, so synaptic currents flowing through each of them could be of hundreds of micro-amps. On the other hand, compact CMOS neurons integrate synaptic current pulses on small capacitors in the range of tens to hundreds of femto-farads, thus presupposing current pulses in the range of few nano-amps, pico-amps or even less.

This problem is not normally highlighted in the literature. Researchers have often performed electrical measurements and characterizations on isolated memristor devices or crossbars and then extrapolated their extracted models to numerically simulated full systems [3–5], circumventing the physical problem of current scaling. In other studies, memristor crossbar currents are sensed by on-chip analogue-to-digital converters [6,7], or driven off-chip and integrated by analogue integrators with operational amplifiers and large off-chip capacitors [8]. Other reported solutions downscale the memristor current in each synaptic circuit, resulting in costly area overheads [9], or use the memristor crossbar as a digital memory, reading it out with sense amplifiers and using the read digital words to activate correctly scaled, dedicated, digitally controlled current injectors [10].

Here we propose a solution based on current splitting using compact MOS ladder circuits [11], inserted between the memristor crossbar and the analogue CMOS neurons, with one splitter circuit per neuron. We also built a 1T1R memristor crossbar by combining isolated custom-made special high-on-resistance memristors, available on custom chips, with NMOS selector transistors fabricated in CMOS technology. This method was used to assemble a full analogue memristor + CMOS multi-chip system. We demonstrate this system’s functionality with examples of its use in winner-takes-all (WTA) one-shot training and stochastic binary (SB) spike timing-dependent plasticity (STDP) learning [12,13]. In summary, the contributions/innovations in the present paper are the following:

— **Ladder circuit:** the use of one ladder circuit per neuron allows for an efficient, low power and low area means of interfacing memristor synapses and CMOS neurons (§§2–3).
— **Modified neuron circuit:** we present a modification of a previously reported neuron circuit that allows to tune its threshold voltage, depending on the application (§4).
— **New memristor stack:** in this paper we use a new memristor stack (with Ti/C/Au top electrode) aimed at reducing the set/reset voltage to below 3V (§5), while providing an OFF resistance in the mega-ohm range.
— **Hybrid Memristor-CMOS multi-chip architecture:** we present a new practical low-cost set-up for interfacing custom-lab made memristors with 1T MOS transistors made with mainstream CMOS technologies (§6).

## 2. The problem of big differences in current domains

Most currently available memristor devices have low resistance states (LRS) in the range of 1–10 kilo-ohms from about $R_{\mathrm{ON}}≃2\;k\Omega$ to about 20 kΩ, and high-resistance-states ($R_{\mathrm{OFF}}$) typically above 100 kΩ but with higher degrees of variability [14]. When these memristors are used in a crossbar configuration for performing computational inference (for example, a vector–matrix multiplication) each active memristor is subject to a relatively small amplitude read pulse in the range of $V_{\mathrm{Read}}≃100\;\mathrm{mV}-300\;\mathrm{mV}$, to avoid alteration of the stored resistance state. This read pulse is typically applied for a time $T_{P}$ in the range of hundreds of nano-seconds or a few micro-seconds. As a result, the charge packet delivered by an individual LRS memristor when stimulated by one single read pulse could be in the order of

2.1 $$\begin{array}{rl} & \delta q_{\mathrm{memr}}=I_{\mathrm{ON}}\times T_{P}≃50\;\text{μ}A\times 100\;n\;s=5\;\mathrm{pC}\\ & \text{with}\;I_{\mathrm{ON}}=V_{\mathrm{amp}}/R_{\mathrm{ON}}≃100\;\mathrm{mV}/2\;k\Omega =50\;\text{μ}A. \end{array}$$

The post-synaptic neurons on the other side of the memristive crossbar integrate all the charge packets produced by dynamically arriving spiking input patterns. When implemented on-chip, these neurons should have minimum area. To properly recognize complex features, they also need to integrate spikes coming from a large number of synapses. These neurons typically comprise a compact integration capacitor $C_{\mathrm{memb}}$, which integrates the charge packets δq coming from the different synapses. The integration is typically leaky, so incoming synaptic charge packets have to coincide within a time window and allow the integrated capacitor voltage to reach a given threshold voltage $V_{\mathrm{th}}$ fast enough to counteract the leakage. When the neuron reaches this threshold, its capacitor voltage is reset to the resting level $V_{\mathrm{rest}}$. As a rule of thumb, a neuron in a large-scale neural network can on average be expected to fire after receiving between a hundred and a thousand incoming spikes. This means that the increment or decrement induced in the integrating capacitor’s voltage by a single ‘average’ synaptic spike should be about ${\Delta V}_{\mathrm{spk}}≃(V_{\mathrm{th}}-V_{\mathrm{rest}})/n_{\mathrm{spk}}$ with $n_{\mathrm{spk}}$ typically in the range of $10^{2}$ to $10^{3}$. In analogue CMOS neurons $V_{\mathrm{th}}-V_{\mathrm{rest}}$ is typically in the range of 1 V [15], so ${\Delta V}_{\mathrm{spk}}$ would be a round 1 mV to 10 mV. For compact CMOS neurons, capacitance $C_{\mathrm{memb}}$ should be kept around 100 fF or less. Therefore, one individual synaptic charge packet $\delta q_{\mathrm{neur}}$ feeding the membrane capacitance should, on average, satisfy

2.2 $$\delta q_{\mathrm{neur}}≃C_{\mathrm{memb}}\times \Delta V_{\mathrm{spk}}=100\;\mathrm{fF}\times 1\;\mathrm{mV}=0.1\;\mathrm{fC}.$$

This charge packet is about a five orders of magnitude smaller charge packet than in equation (2.1). Even for smaller scale proof-of-concept systems with $n_{\mathrm{spk}}$ in the order of 10–100 and $\Delta V_{\mathrm{spk}}≃100\;\mathrm{mV}$, we would still need to scale the charge packets down by about 3 orders of magnitude. One possibility is to use very fast read pulses with $T_{P}$ in the range of 100 ps or less. This would require the use of fast deep-submicrometre technologies and fast crossbar driver circuits. Alternatively, one may think of using a $10^{3}-10^{5}$ larger capacitor, but this implies multiplying by the same factor its area, making it prohibitive (for example, in our neuron design as explained later, $C_{\mathrm{memb}}$’s area is 20% of the neuron area; increasing it $10^{3}$ times would increase the neuron area by about 200 times). If very fast pulses are not feasible, the only alternative is to provide some kind of mechanism for scaling or mapping from the memristor-domain current/charge-packet levels to the neuron-domain current/charge-packet levels.

One solution is to use ADC converters to collect the memristor crossbar currents [6,16,17]. The information is then switched to the digital domain, where the rest of the computation can be performed. Alternatively, other researchers have proposed techniques to scale from the memristor-domain to the neuron-domain current levels inside each synaptic circuit [9]. This, however, results in a high overall chip area penalty. In this study, we decided to use a compact ladder-based circuit at each neuron input, capable of scaling down the memristor-domain current by several orders of magnitude. This also results in a highly energy-efficient technique, due to circuit simplicity, as will be highlighted later in the experimental results (table 5). For now, let us define the energy consumption by one LRS memristor as $E_{\mathrm{LRS}}=V_{\mathrm{DD}}\times \delta q_{\mathrm{memr}}$, where $V_{\mathrm{DD}}$ is the power supply voltage.

## 3. Compact ladder-based circuit for current downscaling

**Figure 1.**
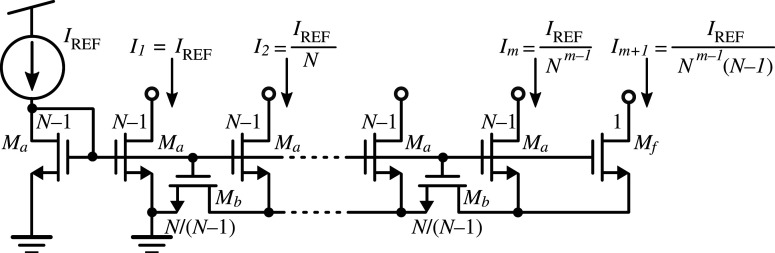
Circuit schematic for generic current splitting ratio N.

MOS-based ladder circuits have been known since 1992 [11] and have proven capable of downscaling currents from hundreds of micro-amps to a few femto-amps [18]. Figure 1 illustrates the MOS ladder-based current-splitting technique for a generic branch-to-branch scaling factor N. The transistors have a size ratio of either W/L=N−1, W/L=N/N(N−1), or W/L=1. Normally, ladder circuits are used with N=2, thus providing binary-weighted currents which are very convenient in, for example, digital to analogue converters. Here, however, we needed to downscale the current aggressively with a reduced number of transistors. We therefore used current ladders with N=10, while minimizing transistor dimensions.

**Figure 2.**
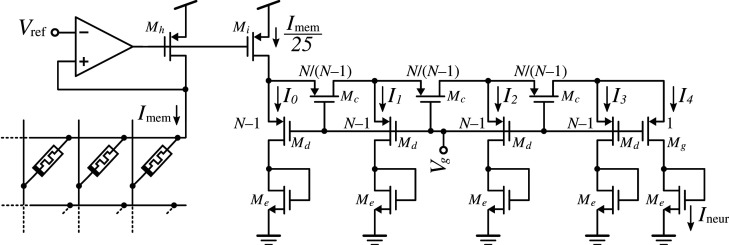
Compact ladder-based MOS current splitter circuit used to downscale the memristor-domain current $I_{\mathrm{mem}}$ (in the range of 10–100 μA) by five orders of magnitude to neuron-domain current levels. Input mirror $M_{h}$–$M_{i}$ provides an extra 25 factor attenuation.

Figure 2 shows the specific circuit used in this study. It used a ladder branch-to-branch scaling factor N=10, with transistor sizes as shown in table 1, downscaling currents by about 5 orders of magnitude from the memristor-domain to the neuron-domain. The area consumed by this atteanuator circuit is $68\times 36\;\text{μ}m^{2}=2448\;\text{μ}m^{2}$. Depending on the scale of the neural network, the average number of synapses connecting to a neuron, the pulse width stimulating the memristors, the value of the memrsistor ON (LRS) resistance, and the desired average number of incoming spikes that should trigger a post-synaptic neuron output spike, one may need a different down-scaling factor for the current attenuator. In our fabricated prototype, we connected branch $I_{4}$ in figure 2 to provide the current for the neuron $I_{\mathrm{neur}}$. However, any other branch could have been selected, from $I_{4}$ to $I_{0}$, to feed current $I_{\mathrm{neur}}$ giving the possibility of scaling down by 5 to one orders of magnitude, respectively.

**Table 1.** PMOS ladder transistor sizes.

	$M_{c}$	$M_{d}$	$M_{e}$	$M_{g}$	$M_{h}$	$M_{i}$
W (μm)	1.1	9	1	1	25	1
L (μm)	1	1	1	1	4	4

## 4. CMOS neuron circuit

**Figure 3.**
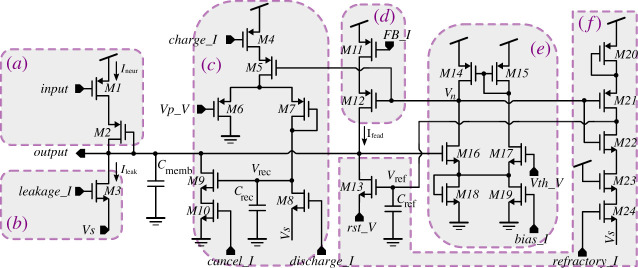
Circuit level schematic of the neuron circuit. (Online version in colour.)

Figure 3 shows the neuron circuit employed in the study. The schematic is separated into six conceptual blocks, A to F. This neuron is a leaky integrate-and-fire neuron, with positive feedback to sharpen spikes, a frequency adaptation mechanism, and a refractory period mechanism. It is based on the CMOS neuron reported by Qiao *et al*. [19] in which we simplified some parts, but added block E so that the neuron threshold voltage can be freely adjusted. Block A feeds the input spikes, block B provides constant leakage, block C provides the frequency adaptation mechanism, block D controls the positive feedback spike-sharpening mechanism, block F provides the refractory mechanism and block E is a comparator that activates blocks C, D and F. Transistor M1 in block A mirrors the output current pulses coming from the current–attenuator $I_{\mathrm{neur}}$ into the neuron circuit, where they are integrated by membrane capacitor $C_{\mathrm{memb}}$. Transistor M2 tends to isolate the neuron from the incoming synaptic circuit during spike generation to minimize crosstalk. Concurrently, transistor M3 in block B introduces a comparatively small but continuous leakage current $I_{\mathrm{leak}}$, which slowly discharges the membrane capacitor. Capacitor $C_{\mathrm{memb}}$’s top terminal (which is the neuron’s output terminal Output) is also connected to the negative input in the comparator (block E). When the Output voltage exceeds the reference voltage Vth_V of the comparator’s positive input, the comparator’s output voltage at node $V_{n}$ starts falling. As a result, transistor M12 activates block D and injects a positive feedback current $I_{\mathrm{fead}}$ into $C_{\mathrm{memb}}$, leading to the generation of a sharper spike at Output. Simultaneously, transistor M5 activates block C, causing a charging current to be injected into recovery capacitor $C_{\mathrm{rec}}$. Block C is in charge of the spike-frequency adaptation mechanism, which serves to progressively lower the neuron’s firing rate in response to a continuous input stimulation [20]. This way, block C adds an additional leakage current to membrane capacitor $C_{\mathrm{memb}}$ when the neuron’s spiking output activity increases. Block F implements the refractory mechanism. Transistor M21 is activated on the rising edge of a spike (falling edge at node $V_{n}$) and charges refractory capacitor $C_{\mathrm{ref}}$. This leads to the discharge of $C_{\mathrm{memb}}$ through M13, in order to hold Output close to rst_V. After a spike, capacitor $C_{\mathrm{ref}}$ is discharged with a small current controlled by voltage refractory_I via transistors M22-M24. The refractory period lasts until $V_{\mathrm{ref}}$ falls below transistor M13’s threshold voltage.

The three capacitances of the neuron circuit in figure 3 were designed with $C_{\mathrm{memb}}=150\;\mathrm{fF}$, $C_{\mathrm{ref}}=100\;\mathrm{fF}$ and $C_{\mathrm{rec}}=100\;\mathrm{fF}$. The total area consumed by the neuron is $57.7\times 15.5\;\text{μ}m^{2}=863\;\text{μ}m^{2}$, including all three capacitors. This CMOS technology allows placing capacitors over the transistors, while their density is about $1\;\text{μ}m^{2}/\mathrm{fF}$. Consequently, the area of the three capacitors is about $350\;\text{μ}m^{2}$ and could be fit above the rest of the neuron circuitry.

## 5. RRAM devices

**Figure 4.**
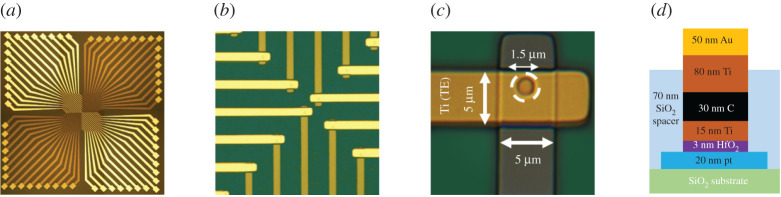
Microphotographs of (*a*) the memristor die, (*b*) the top and bottom interconnects, and (*c*) the memristor active area. (*d*) The RRAM device structure. (Online version in colour.)

In this paper, we report for the first time experimental results using a new memristor device aimed at reducing the set/reset voltages to below 3V, while presenting an OFF resistance of about 1 MΩ or higher. Figure 4*a*–*c* shows SEM images of the RRAM cells used in this study. The RRAM devices comprised stacks are made up of a Pt bottom electrode (BE), a ${\mathrm{HfO}}_{2}$ active layer and a Ti/C/Au top electrode (TE). The fabrication procedure was as follows. First, a ${\mathrm{SiO}}_{2}$ layer was deposited by chemical vapour deposition on a heavily p-doped silicon wafer serving as substrate. The 20 nm thick Pt BE was then deposited using ultra-high vacuum ($10^{-7}\;\mathrm{mbar}$) e-beam evaporation and patterned using lithography and lift-off. All the lithographic steps were performed using a Heidelberg MLA100 UV laser writer. A 70 nm thick ${\mathrm{SiO}}_{2}$ spacer layer was then deposited to isolate the BE lines from the TE lines. Holes with diameters of 1.5 μm were then made through the spacer layer by reactive ion etching. The holes were opened in correspondence of the BEs with regular lattice spacing, to serve as cell active regions in the array. The 3 nm thick ${\mathrm{HfO}}_{2}$ film was then deposited, again by e-beam evaporation, followed by the TE stack, without breaking the vacuum between the different layers. A 15 nm thick Ti cap layer was deposited on ${\mathrm{HfO}}_{2}$, followed by a 30 nm C layer to increase the series resistance and thus reduce the possible overshoot effects at forming and set transitions. A thick Ti/Au layer was finally deposited as an electrical contact. The oxide/TE stack was patterned using lithography and lift-off.

The RRAM device structure can be seen in figure 4*d*. Possible short circuits between the TE and BE layers are prevented by the ${\mathrm{SiO}}_{2}$ spacer layer. The Ti cap layer has been reported to act as an oxygen scavenger, leading to the formation of a TiOx oxygen-exchange layer at the ${\text{Ti-HfO}}_{2}$ interface [21]. This mechanism leads to an increase in the local concentration of oxygen vacancies in ${\mathrm{HfO}}_{x}$, which in turn enhances the leakage current in the pristine state and reduces the devices’ forming voltage [22].

**Figure 5.**
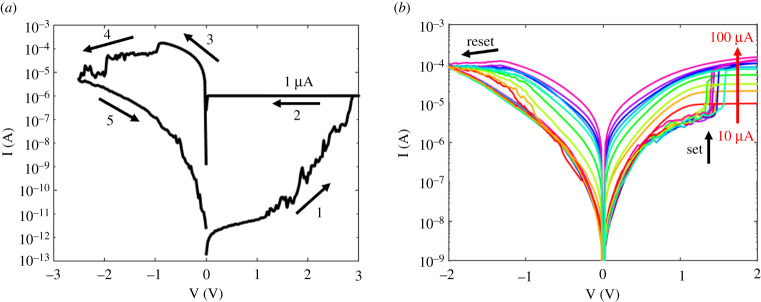
I–V characteristics of the RRAM device. (*a*) Forming characteristics. The device is initialized by the forming operation with a relatively small compliance current of 1 μA (1-2). The first reset operation (3) shows a relatively large reset current of about 150 μA, due to the parasitic currents during the high voltage forming. Extending the reset operation to large negative voltages (4) allows to reach a deep reset state with relatively large resistance (5). (*b*) set/reset characteristics. The following set/reset curves show a relatively tight distribution of set voltages and controllable resistance via the compliance current. (Online version in colour.)

The RRAM devices were first characterized using an Agilent 4156C Precise Semiconductor Parameter Analyzer. Figure 5 shows the multiple-cycle DC characteristics indicating repeatable set and reset transitions when positive and negative voltages, respectively, were applied to the TE. Also shown is the first cycle, with the forming transition taking place at around 3 V. This is relatively high compared with the typical set voltage of about 1.5 V. A curve was collected for each increment in compliance current $I_{C}$ from 10 μA to 100 μA. As $I_{C}$ increased, the LRS conductance increased almost linearly with it, while the high resistance state (HRS) conductance remained almost constant.

**Figure 6.**
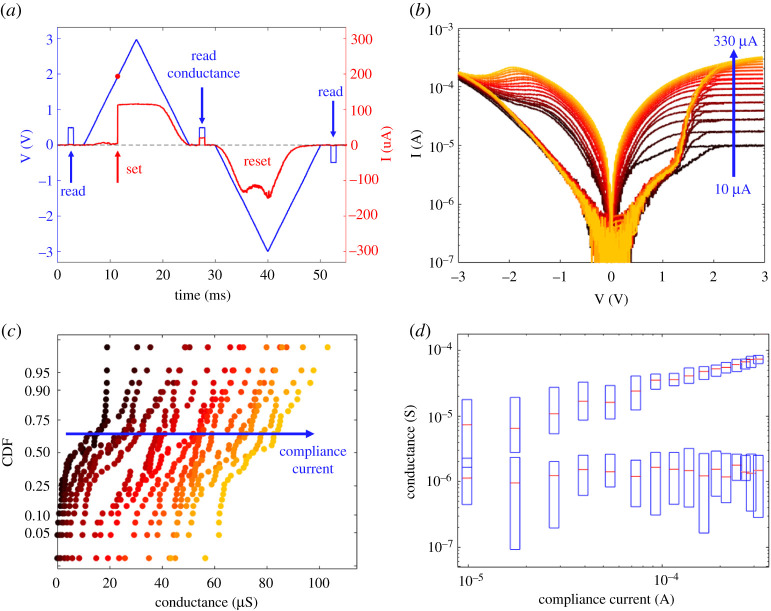
Pulse characterization of the memristors: (*a*) measured current (red) and applied voltage (blue) versus time, (*b*) I–V curves obtained by applying triangular pulses, (*c*) cumulative distributions of the measured conductance, and (*d*) measured HRS and LRS conductance values as a function of compliance current $I_{C}$. (Online version in colour.)

The pulsed operation of the RRAM devices was studied using a TTi-TGA12102 Arbitrary Waveform Generator and a LeCroy WaveRunner 640Zi oscilloscope. The compliance current was applied by an external transistor. Figure 6*a* shows the oscilloscope traces for the applied voltage and the measured current. Positive and negative triangular pulses with pulse-widths of 20 ms were applied for set and reset transitions, respectively. From the measured current and voltage, we obtained the pulsed I-V curves displayed in figure 6*b*. Each curve represents the averaged current between 100 characteristics. The results indicate a nonlinear characteristic where the resistance window increases with $I_{C}$. Figure 6*c* shows the cumulative distributions of the measured LRS conductance G at increasing $I_{C}$, indicating a standard deviation of about 12 μS. Figure 6*d* shows the measured HRS and LRS conductance values as a function of $I_{C}$, supporting the increase in the resistance window at increasing compliance current. Conductance increased with a slope of approximately one, indicating a linear relationship with $I_{C}$, except for the relatively low G, where the LRS collapsed with the HRS level.

For our set-up and experiments described in the rest of this paper, we wanted to have a large resistance window, maximizing the OFF resistance (HRS). We therefore used high compliance current levels and used the memristors as binary memories with maximally separated LRS and HRS.

## 6. System architecture

**Figure 7.**
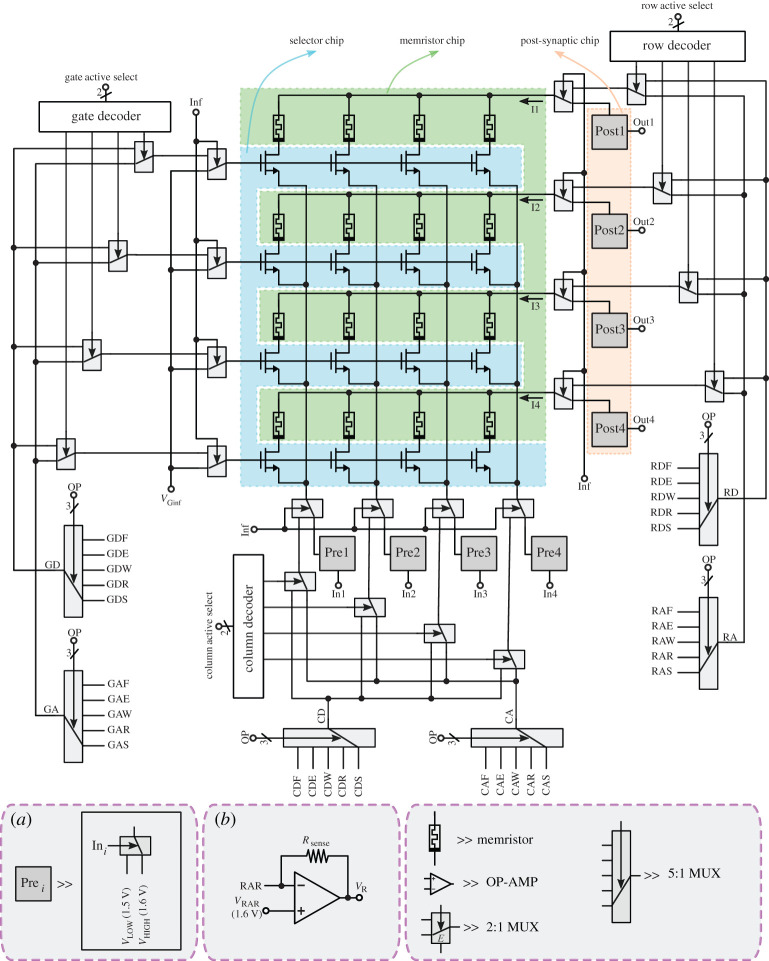
System level architecture of the memristor-based spiking neural network. (Online version in colour.)

In this paper, we showcase a small 4×4 1T1R synaptic memristor crossbar with CMOS analogue neurons performing learning and inference. Our system illustrates how to use a set of separate CMOS chips together with custom made memristive devices to build a hybrid CMOS–memristor system operated with an auxiliary custom PCB and controlled by an FPGA. The interesting novelty about this approach is that it shows a simple way for memristor researchers to assemble 1T1R arrays by combining custom memristor-only chips with standard CMOS ASICs. The system’s overall architecture is shown in figure 7. The memristors were on a separate chip (coloured green in figure 7). As can be seen in figure 4*a*, a total of 32 separate memristors were on the chip. Owing to yield issues, however, not all 32 were functional. Here we used a total of 16 memristors, each having a separate pin for its bottom plate while the top plates were shared by the four memristors in the same row (figure 7). The 16 NMOS selectors were fabricated on a separate CMOS chip (coloured blue in figure 7). These shared their gates row-wise and their source terminals column-wise. Their drains were connected individually to each of the memristors’ bottom plates. This way, a full 4×4 1T1R synaptic array could be assembled using custom memristors.

The post-synaptic CMOS circuits (shown in orange in figure 7) were allocated on a separate chip. They each included one current attenuator (§3) and one CMOS neuron circuit (§4). The other elements in figure 7 were allocated on a custom PCB. This custom PCB had some external digital control signals (‘Row Active Select’, ‘Column Active Select’ and ‘Inf’), set by an additional FPGA control board running a state machine. The system in figure 7 could be configured in two operation modes, ‘Inference’ mode or ‘Element-Wise’ mode, using a digital control signal Inf.

### (a) Inference mode

When Inf=1 the inference mode is activated and the synaptic crossbar will perform parallel inference. In this mode, all 1T gates are connected to a gate inference voltage bias $V_{\mathrm{Ginf}}$, each of the four memristor row top plates is connected to one post-synaptic circuit, and each of the four 1T source columns is connected to one pre-synaptic circuit (${\mathrm{Pre}}_{i}$ in figure 7). The pre-synaptic circuits are physically implemented on the custom PCB and are simple switches connecting the column to either a $V_{\mathrm{HIGH}}$ or $V_{\mathrm{LOW}}$ voltage, depending on digital input ${\mathrm{In}}_{i}$ (either ‘0’ or ‘1’), as illustrated in the inset in figure 7. In this ‘Inference Mode’, all post-synaptic rows are set to voltage $V_{\mathrm{HIGH}}$, while active digital input ${\mathrm{In}}_{i}$ will set the corresponding column to $V_{\mathrm{LOW}}$. This way, the current flowing from a post-synaptic circuit will be given by

6.1 $$I_{j}=\sum_{i=1}^{4}R_{ij}^{-1}\Delta V_{\mathrm{Read}}{\mathrm{In}}_{i},$$

where $R_{ij}$ is the resistance of the 1T1R synapse at column i and row j, and $\Delta V_{\mathrm{Read}}=V_{\mathrm{HIGH}}-V_{\mathrm{LOW}}$. In this ‘Inference Mode’, post-synaptic currents $I_{j}$ will be collected by the current attenuator circuits and scaled down before being integrated in the neurons. The neuron outputs ${\mathrm{Out}}_{j}$ are monitored by the FPGA state machine.

### (b) Element-wise mode

When Inf=0, the ‘Element-wise Mode’ is activated. In this mode, only one column and only one row at a time are set as ‘active’, so only one 1T1R element is selected to perform an individual ‘Forming’, ‘Write’, ‘Erase’ or ‘Read’ operation on them. The 2-bit digital control ‘Row Active Select’ sets one of the rows as the active row, while the 2-bit digital control ‘Column Active Word’ sets one of the columns as the active column. The other, non-active, rows and columns are set as ‘default’. For the active row, the gates of the 1T NMOS selector transistors are connected to node ‘GA’ (Gate Active) while the other gates are connected to node ‘GD’ (Gate Default). The memristor top plate node of the ‘active’ row is connected to node ‘RA’ (Row Active), while the others are connected to node ‘RD’ (Row Default). Finally, the active column is connected to node ‘CA’ (Column Active), and the other columns are connected to node ‘CD’ (Column Default).

The operation to be performed at the individually selected 1T1R synapse is set by the 3-bit digital control word ‘OP’. It may be a ‘Forming’, ‘Write’, ‘Erase’, ‘Stand-by’ or ‘Read’ operation. The stand-by mode is ‘Read-Mode’, in which all terminals are connected to the ‘default’ value. This is a safeguard measure to avoid undesirable glitches when switching active rows/columns or operation modes. Stand-by mode should therefore be inserted when switching between ‘Forming’, ‘Write’, ‘Erase’ or ‘Read’ operations. Similarly, stand-by mode should also be used when changing active columns/rows. It should also be noted that the ‘Forming’, ‘Write’, ‘Erase’ and ‘Read’ operations are to be performed during well-defined time durations, while ‘Stand-by’ can have an arbitrary duration.

For each of the six modes in figure 7, GA (Gate Active), GD (Gate Default), RA (Row Active), RD (Row Default), CA (Column Active) and CD (Column Default) the corresponding active lines should be connected to five different bias voltages, depending on the selected operation. This results in a total of 30 different bias voltages, each of which can be adjusted individually on the custom PCB. These 30 bias voltages are available at the 30 nodes in figure 7 which are labelled with three capital letters XYZ , where ‘X’ is either ‘G’ (Gate), ‘R’ (Row), ‘C’ (Column), ‘Y’ is either ‘A’ (Active) or ‘D’ (Default), and ‘Z’ is either ‘F’ (Forming), ‘E’ (Erase), ‘W’ (Write), ‘R’ (Read) or ‘S’ (Stand-by).

**Figure 8.**
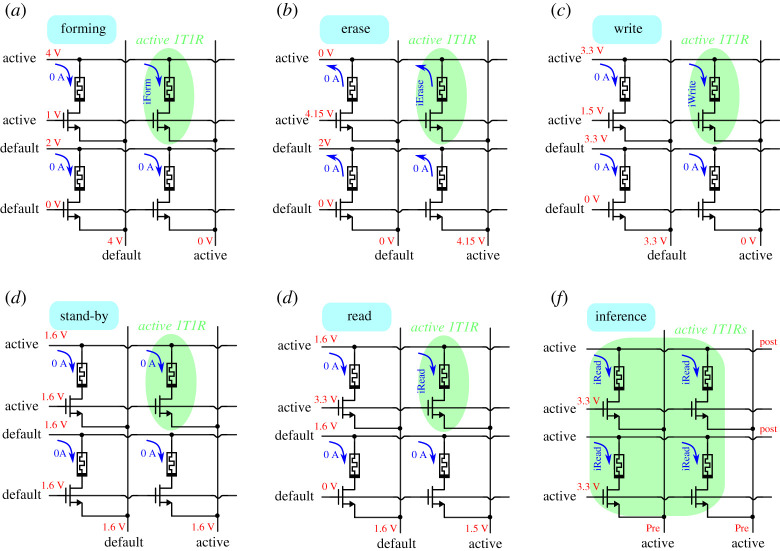
Illustration of the ‘active’ and ‘default’ voltage levels used for the different operating modes. (*a*) Forming, (*b*) erase, (*c*) write, (*d*) stand-by, (*e*) read and (*f*) in the parallel inference mode. (Online version in colour.)

Figure 8 indicates the gate, column and row voltages to be set for the active and default columns and rows for the five different operation modes and the inference mode.

These voltage bias settings are summarized in table 2. During element-wise reading, the aim is to accurately read the resistance of the selected 1T1R synapse. To do this, the current sensing-circuit shown in inset (*b*) in figure 7 is connected to node RAR. By reading voltage $V_{R}$, it is possible to infer the resistance of the corresponding 1T1R synapse by using

6.2 $$R_{ij}=R_{\mathrm{sense}}\frac{V_{\mathrm{HIGH}}-V_{\mathrm{LOW}}}{V_{R}-V_{\mathrm{RAR}}}=R_{\mathrm{sense}}\frac{0.1\;V}{V_{R}-1.6\;V}.$$

**Table 2.** Active and default bias voltages used for the different operations.

		element-wise mode					
		forming	erase	write	read	stand-by	inference-mode
gate	active	1 V	4.15 V	3.3 V	1.6 V	1.6 V	3.3 V
	default	0 V	0 V	0 V	0 V		
row	active	4 V	0 V	3.3 V	1.6 V	1.6 V	1.6 V
	default	2 V	2 V	3.3 V	1.6 V		
column	active	0 V	4.15 V	0 V	1.5 V	1.6 V	1.6 V / 1.5 V
	default	4 V	0 V	3.3 V	1.6 V		

The sizing of the selector transistor is critical. On one hand, it is desirable it is not too large. This way, when scaling up the system, higher synapse densities can be achieved. However, on the other hand, its size ratio (W/L) should be large enough for allowing the maximum required currents for the different operations. The most critical operation is resetting the memristor from LRS to HRS, since the memristor has a low resistance while we need to apply to it a relatively large voltage of about 3 V. We sized our selector transistor to have minimum length (L=350 nm) and a width of W=13.4 μm. Under these conditions, we could erase safely all fabricated and operational memristors while applying an erase voltage to the 1T1R compound of 4.15 V, as shown in table 2.

## 7. Experimental results

Three separate chips were fabricated. One was a custom memristor chip containing 32 individual memristors and the other two were ASIC chips fabricated in TSMC 180 nm CMOS technology, one of which contained the NMOS transistor selectors and the other the CMOS current attenuators and neurons for the post-synaptic circuits.

**Figure 9.**
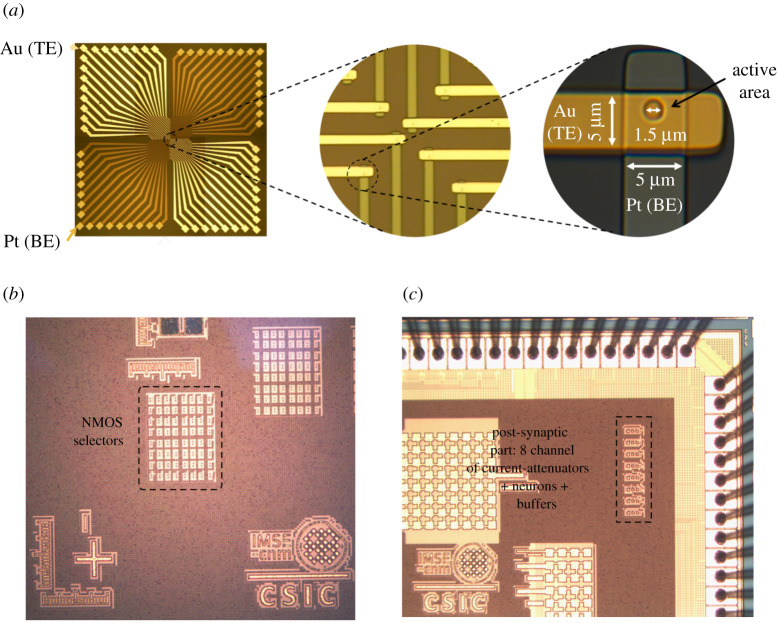
Micrographs of (*a*) the memristor chip (with progressive zoom-ins), (*b*) the selector transistor chip, and (*c*) the post-synaptic circuit chip. (Online version in colour.)

Figure 9 shows micrographs of the three chips. Details of the custom memristor chip can be seen in figure 9*a*. This chip has a total of 32 independent, individual memristors, each connected to an Au (gold) top electrode and a Pt (platinum) BE. Each electrode is 5 μm wide and the circular active area of each memristor at the cross points on both electrodes has a diameter of 1.5 μm. The chip has a total of 64 pins (32 TE pins and 32 BE pins). Not all of the 32 memristor devices were fully functional. From the ones that were, 16 were selected to be connected to the CMOS chips.

Figure 9*b* shows a micrograph of the fabricated NMOS selector chip. Each NMOS transistor has a size of W=6.7 μm and L=350 nm. The fabricated array has 8×8 selectors. Since we used only 16 memristors, a sub-array of 4×4 selectors was used in the set-up. Figure 9*c* shows a micrograph of the post-synaptic chip. It includes a total of eight post-synaptic circuits, each including one current attenuator, one CMOS neuron and one output buffer. Each post-synaptic circuit has an area of $180\times 50\;\text{μ}m^{2}$, of which $2520\;\text{μ}m^{2}$ are occupied by the current-attenuator, $840\;\text{μ}m^{2}$ by the neuron and $1272\;\text{μ}m^{2}$ by the buffer. In our set-up, four post-synaptic circuits were used.

**Figure 10.**
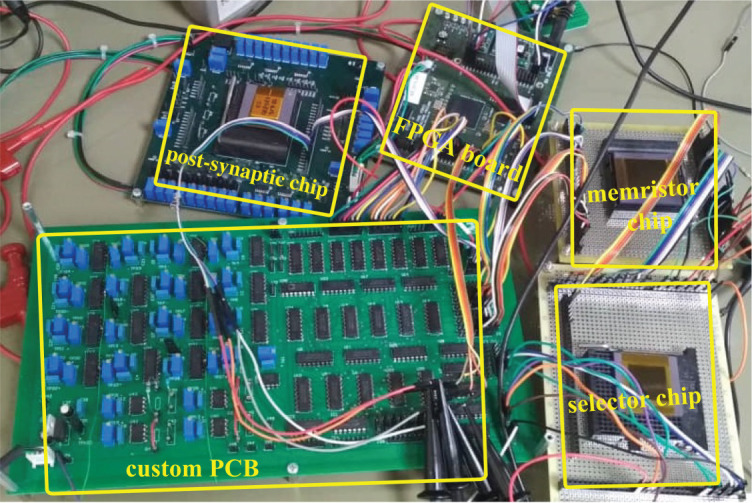
Experimental test set-up showing all PCBs and chips. (Online version in colour.)

Figure 10 shows the full experimental set-up, including one PCB holding the memristor chip, another PCB holding the selector chip, another PCB holding the post-synaptic circuit chip, the custom PCB with all the switches, multiplexers and potentiometers providing all the biases and the FPGA-based controller PCB.

### (a) Current-attenuator test

**Figure 11.**
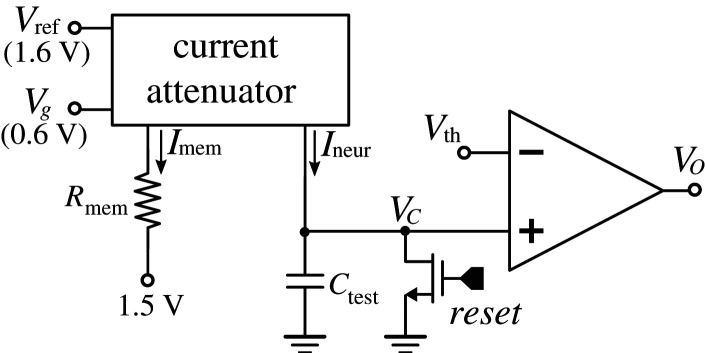
On-chip dedicated circuit used to characterize the current attenuator output current.

Figure 11 shows the on-chip dedicated circuit used to characterize the current attenuator. Since the attenuator output currents can be very small (less than 1 pico ampere), currents cannot be driven off-chip and measured by external instruments [18]. With the circuit in figure 11, it was possible to measure the charging time of the integrating capacitor and estimate the charging current coming from the attenuator output $I_{\mathrm{neur}}$. The current attenuator input current $I_{\mathrm{mem}}$ was set by changing the off-chip resistance $R_{\mathrm{mem}}$.

7.1 $$I_{\mathrm{mem}}=\frac{100\;\mathrm{mV}}{R_{\mathrm{mem}}}.$$

To measure the attenuated output current $I_{\mathrm{neur}}$, capacitor $C_{\mathrm{test}}$ was initially discharged to 0 V and then charged by $I_{\mathrm{neur}}$ until the capacitor voltage reached $V_{\mathrm{th}}$. By observing the digital output $V_{o}$ of the voltage comparator, it was possible to measure the time Δt between capacitor reset and the instant at which the capacitor voltage reached $V_{\mathrm{th}}$

7.2 $$I_{\mathrm{neur}}=\frac{C_{\mathrm{test}}V_{\mathrm{th}}}{\Delta t}.$$

Capacitor $C_{\mathrm{test}}$ was designed with a capacitance of 1.5 pF and voltage $V_{\mathrm{th}}$ was set at 500 mV.

**Figure 12.**
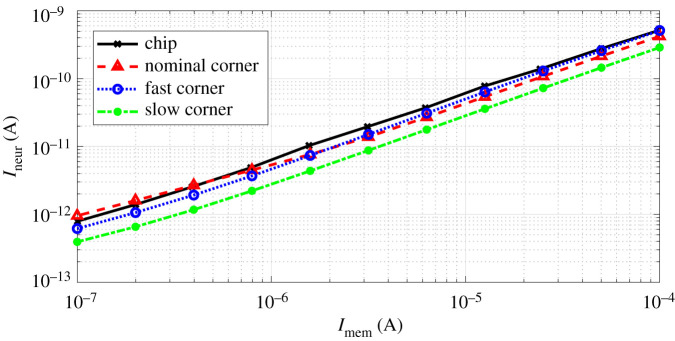
Current-attenuator output current $I_{\mathrm{neur}}$ versus its input current $I_{\mathrm{mem}}$. (Online version in colour.)

Figure 12 shows the characterization results for the measured values of current $I_{\mathrm{neur}}$ versus the attenuator input current $I_{\mathrm{mem}}$. It can be seen that the attenuation factor for synaptic memristances $R_{\mathrm{mem}}$ in the range of 1 kΩ to 10 kΩ was between $1.9\times 10^{5}$ and $1.6\times 10^{5}$. Table 3 summarizes the values used for $R_{\mathrm{mem}}$, the values measured for Δt, and the inferred values for $I_{\mathrm{mem}}$, $I_{\mathrm{neur}}$ and the attenuation factor.

**Table 3.** Input ($I_{\mathrm{mem}}$) and output ($I_{\mathrm{neur}}$) currents of the attenuator along with the measured charging times (Δt) at capacitor $C_{\mathrm{test}}$ and the resulting current attenuation factors for a set of different equivalent input memristor resistances $R_{\mathrm{mem}}$.

no.	$R_{\mathrm{mem}}\;(k\Omega )$	Δt (ms)	$I_{\mathrm{mem}}$	$I_{\mathrm{neur}}$	attenuation ($10^{5}$)
**1**	1.02	1.47	97.7 μA	50.86 pA	1.92
**2**	2	2.70	50.0 μA	27.73 pA	1.80
**3**	4.13	5.41	24.2 μA	13.83 pA	1.74
**4**	7.97	9.65	12.5 μA	7.77 pA	1.61
**5**	16.1	20.23	6.21 μA	3.70 pA	1.67
**6**	32	38.34	3.12 μA	1.95 pA	1.59
**7**	64	72.75	1.56 μA	1.03 pA	1.51
**8**	127.7	153.99	783.0 n A	487.03 fA	1.60
**9**	256.1	295	390.4 n A	254.23 fA	1.53
**10**	500	537.59	200 n A	139.51 fA	1.43
**11**	997	947.68	100.3 nA	79.14 fA	1.26

### (b) Neuron circuit test

**Figure 13.**
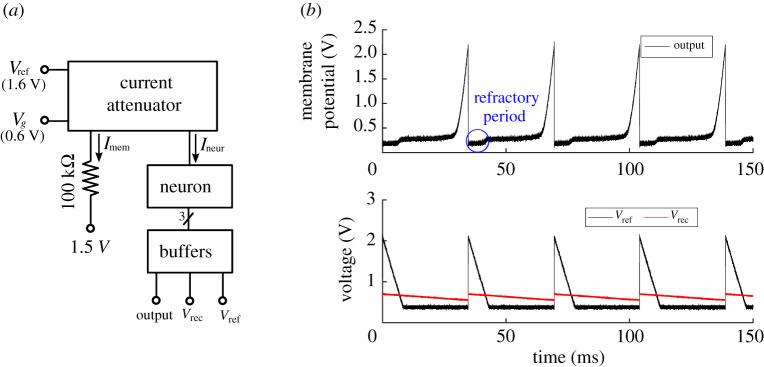
(*a*) Test configuration for the neuron circuit, (*b*) membrane potential (Output), refractory variable voltage ($V_{\mathrm{ref}}$), and recovery variable voltage ($V_{\mathrm{rec}}$) of the neuron circuit. (Online version in colour.)

Figure 13*a* shows the test configuration used to characterize the neuron cell circuit. In this arrangement, an external 1 kΩ resistor was connected between the input node of the current-attenuator and a 1.5 V reference voltage. For this resistance, it can be seen from table 3 that the attenuator output current to the neuron $I_{\mathrm{neur}}$ was slightly below 1 pA. Three buffers were used to isolate the three capacitor voltages in figure 13*a* (Output, $V_{\mathrm{ref}}$ and $V_{\mathrm{rec}}$) and thus allow efficient, undisturbed off-chip observation. Figure 13*b* shows these three neuron voltages during a time period of 150 ms. In this experiment, the neuron threshold voltage was set to 2.2 V and the refractory period was set to about 7 ms.

### (c) One shot WTA training

**Figure 14.**
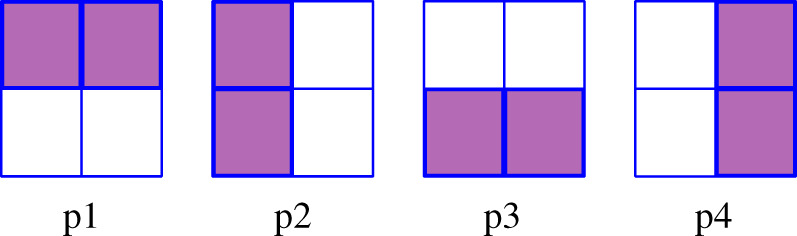
Input patterns to the SNN. (Online version in colour.)

In a first system-level experimental set-up, we performed a one-shot WTA-driven training demonstration. For this purpose, all 16 memristors in the crossbar were first initialized to their ON state (LRS). The four 4-bit patterns p1, p2, p3, p4 shown in figure 14 were then used as input patterns. For each pattern, simultaneous pulses were applied by the pre-synaptic neurons with an active bit. The patterns were applied for long enough to allow at least one of the output neurons to reach its threshold level. The neuron that first reached its threshold level was the winning neuron, and the weights of the synapses connecting to it were updated. For a synapse between the winning post-synaptic neuron and an active pre-synaptic neuron, no action was taken. For a synapse between the winning post-synaptic neuron and an inactive pre-synaptic neuron resistance was set to high (HRS) by performing an erase operation.

**Figure 15.**
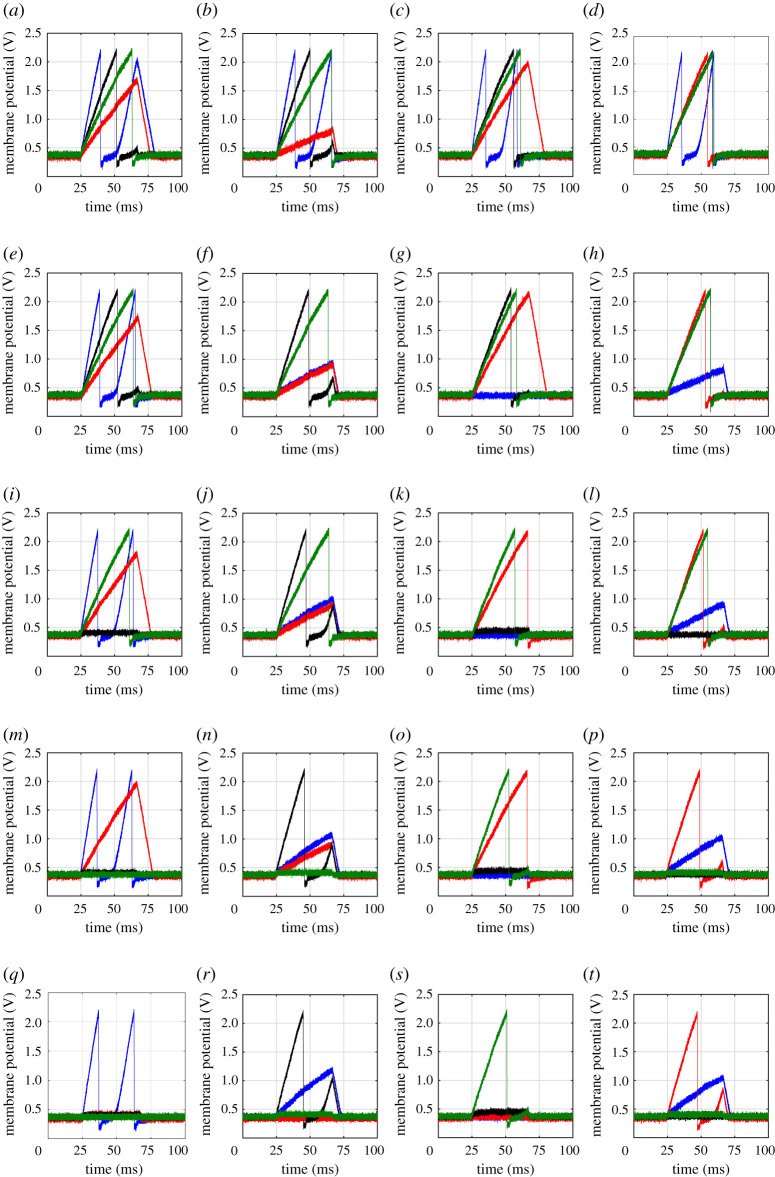
Neuron outputs for each input pattern. (*a*–*d*) before any weight update, (*e*–*h*) after presenting p1 and corresponding weight updates, (*i*–*l*) after presenting p1 and p4 and corresponding weight updates, (*m*–*p*) after presenting p1, p4 and p3 and corresponding weight updates, (*q*–*t*) after presenting all the input patterns and all corresponding weight updates. Blue circles, neuron1; red circles, neuron2; black circles, neuron3; green circles, neuron4. (Online version in colour.)

Figure 15 shows the experimentally measured membrane voltages of the post-synaptic neurons when the input patterns in figure 14 were applied sequentially and the weights updated in accordance with this WTA-driven training method. The left-most column corresponds to input pattern p1, the second left-most column to p2, and so on. The top row (*a*–*d*) shows the measured membrane voltages of the four post-synaptic neurons for each input pattern when all memristors were initially set to LRS. The second row (*e*–*h*) shows the membrane voltages after implementing weight changes when pattern p1 was applied and post-synaptic neuron 1 (blue) won the WTA competition. Therefore, only connections to neuron 1 get updated. Note that when applying input patterns 2 to 4 (see (*f* –*h*)), neuron 1 behaves differently than in (*b*–*d*), since its input weights have changed. The third row (*i*–*l*) shows the voltages after the next input pattern p2 was applied and post-synaptic neuron 3 (black) won. Now, neuron 3 changes its behaviour in (*i,k,l*) with respect to (*e,g,h*). The fourth row (*m–p*) shows the voltages after the next pattern p3 was applied and post-synaptic neuron 4 (green) won. Now neuron 4 changed its behaviour in (*m,n,p*) with respect to (*i,j,l*). Finally, the fifth row (*q–t*), shows the voltages after the next input pattern p4 was applied and post-synaptic neuron 2 (red) won, showing a different behaviour in (*q–s*) with respect to (*m–o*). In this last row, it can clearly be seen that each output neuron responded very strongly to only one of the input patterns.

### (d) Stochastic binary STDP

STDP [23] is a bioinspired learning rule for SNNs and allows, in principle, for continuous on-line learning. SB-STDP is a variant of the original STDP learning rule in which synapses only present an ON and an OFF state and the weight updates follow a stochastic rule [12,13]. Therefore, SB-STDP is quite appropriate for binary RRAM synapse SNNs. Originally, SB-STDP was proposed by simply substituting the original deterministic STDP gradual update [23] by a non-gradual stochastic one [12]. However, later on, it was shown that for correct operation on scaled-up systems some regularization techniques were required [13]. In the example case, we are considering here, which is just a small 4×4 crossbar, we only needed to consider one regularization technique, namely homeostasis.

**Figure 16.**
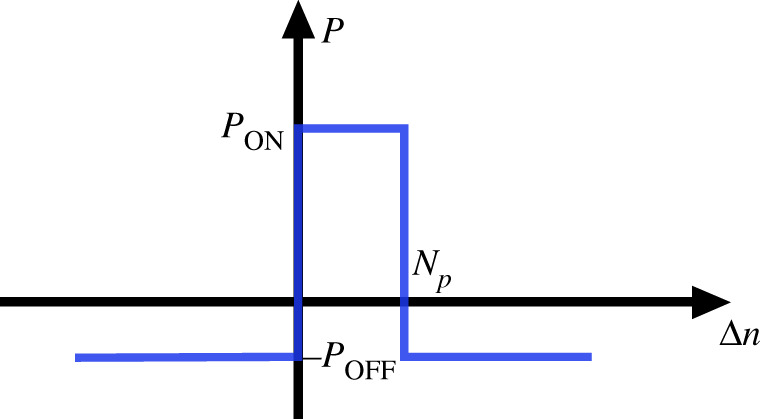
Weight change probability for SB-STDP. If the sequence order between post-synaptic and pre-synaptic spikes is positive and less than $N_{p}$, the corresponding synapse is changed to ON with probability $P_{\mathrm{ON}}$. Otherwise, all other synapses connecting to this post-synaptic neuron are set to OFF with probability $P_{\mathrm{OFF}}$. (Online version in colour.)

In SB-STDP, the synapses connected to a firing post-synaptic neuron are updated following the process explained below with reference to figure 16. The most recent $N_{P}$ input spikes are kept on a list. Input and output spikes are all indexed sequentially by a counter n. Whenever an output neuron j spikes, all previous $N_{P}$ input spikes are retrieved. For each input neuron i, only its most recent spike is considered, so a synapse ij between an active input neuron i on the list and active output neuron j is updated only once for each output spike. If synapse ij is already ON, it is left untouched. But if it is OFF, it is changed to fully ON with a probability $P_{\mathrm{ON}}$. Once all active connections obtained from the list have been updated probabilistically, the total number of ON synapses is counted. In SB-STDP, to implement homeostasis, the sum of ON synapses connecting to an output neuron j is kept constant. Let us call this constant M. If the sum is greater than M, then one of the synapses which was not retrieved from the list and is ON is chosen randomly and its weight is set to OFF. This process is repeated until the sum of ON synapses is M.

**Figure 17.**
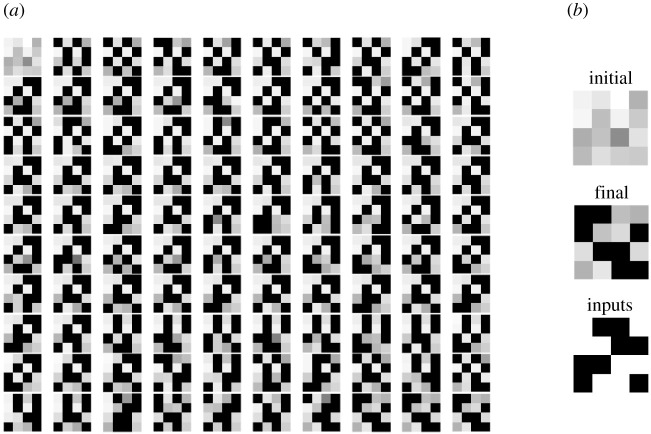
(*a*) Samples of the sequence of weight updates in SB-STDP, (*b*) initial and final weights, and the four (row-wise) input patterns used for SB-STDP training.

To perform SB-STDP in our set-up, we used the four input patterns shown in figure 17*b* bottom, where each input pattern is a horizontal 4-bit row. Each input pattern was applied by having its corresponding active input neurons present a sequence of randomly spaced spikes. Initially, all synaptic memristors were initialized to their ON state (LRS). As soon as an output neuron spiked, the memristors connecting to it were updated following the SB-STDP rule described above. All neurons were then reset, and a new input pattern was applied. Figure 17*a* shows samples of a sequence of weight updates, starting with the initial set of weights (all ON, top left) and finishing with the stabilized set of weights (bottom right). The number iterations until convergence varied from about 15 up to 200, with an average of about 50. The ON weights fall approximately in the 2−6 kΩ range and are shown in grey scale, the minimum resistance in all 16 memristors over the full learning sequence being white. Figure 17*b* shows the initial weights (top), the final weights (centre) and the input patterns (bottom). Each row in figure 17*b* bottom corresponds to one 4-bit input pattern. It can be seen that the final weights correspond to the input patterns but are shuffled row-wise. Consequently, the system successfully learned the input patterns.

## 8. Benchmarking for energy efficiency

In order to compare with other reported state-of-the-art neural processing chip systems, let us first analyse which would be the optimum settings for a chip following our approach to minimize energy consumption per synaptic computation. The largest currents in our system are currents flowing through memristors which are at LRS. Therefore, the minimum energy per operation would be given by finding the fastest pulses that can be applied to LRS memristors while guaranteeing that the circuit providing current to them (transistor $M_{h}$ in figure 2 and the differential amplifier) can propagate the corresponding memristor-domain charge packet down to the neuron-domain with sufficient integrity. By assuming an average LRS value of 10 kΩ, we found (by simulations) this limit to be $T_{p}≃100$ ns, in which case the memristor-domain charge packet was found to be 0.68 pC (about 30% below the ideal value), and the circuit needed a total time of 360 ns to settle. Under these conditions, we analysed which would be the neuron-domain charge packets if selecting for $I_{\mathrm{neur}}$ the different ladder branches in figure 2, from $I_{0}$ to $I_{4}$. To find these charge packets we obtained the voltage increments $\Delta V_{\mathrm{spk}}$ induced at the neuron membrane capacitance $C_{\mathrm{memb}}$. From these voltage increments, we can compute the neuron-domain charge packets as $\delta q_{\mathrm{neur}}=C_{\mathrm{memb}}\times \Delta V_{\mathrm{spk}}$. The results are summarized in table 4. For maximum speed pulses of 100 ns stimulating the memristors, one can observe voltage increments at the neuron membrane voltage when selecting ladder branches $I_{0}$ to $I_{3}$. When selecting branch $I_{4}$, no change is appreciated. Depending on the selected ladder branch, the effective charge packet attenuation ranges from 25.2 up to $1.7\times 10^{4}$. Also, for practical charge packet sizes (those that induce membrane voltage increments in the range of 1 mV–50 mV, as discussed in §2), one would require attenuation factors in the range of hundreds to thousands for such fast 100 ns stimulation pulses.

**Table 4.** Effective charge-packet attenuation for maximum speed and minimum energy.

attenuator branch	$\Delta V_{\mathrm{spk}}$	$\delta q_{\mathrm{neur}}$	effective attenuation
$I_{0}$	183 mV	27 fC	25.2
$I_{1}$	19.5 mV	2.9 fC	234
$I_{2}$	1.88 mV	0.28 fC	$2.43\times 10^{3}$
$I_{3}$	268 μV	0.04 fC	$1.7\times 10^{4}$
$I_{4}$	—	—	—

**Table 5.** Comparison with some reported state-of-the-art neural processing chip systems.

	TrueNorth [24]	Loihi [25]	LETI [10]	Yao [6]	This work
technology	28 nm	14 nm	130 nm	130 nm	180 nm
network type	SNN	SNN	SNN	CNN	SNN
weight storage	SRAM	SRAM	RRAM	RRAM	RRAM
$E_{\mathrm{SOP}}$	27 pJ	105 pJ${}^{a}$	180 pJ	91 pJ${}^{b}$	4 pJ${}^{a}$^,^${}^{c}$

${}^{a}$Energy consumption of communication circuits not included.

${}^{b}$Most of energy consumption is due to peripheral analogue-to-digital converters.

${}^{c}$All memristors are assumed to be at minimum resistance (LRS), thus consuming maximum power.

Regarding energy consumption in our system, there is energy due to stand-by power and energy due to memristor currents and circuit transients during input spikes. The power consumption of the neurons is negligible. A neuron firing at a high rate of 1 KHz consumes about 140 nW. The circuit component consuming most of the stand-by power is the differential amplifier in figure 2, which consumes about 15 μW each. During a synaptic event, the dominant currents are flowing through LRS memristors. The currents flowing through the ladder branches are little fractions of these currents, and may affect also the ratio $E_{\mathrm{LRS}}/E_{\mathrm{SOP}}$. In our 4×4 crossbar system, when setting all memristors to LRS and feeding all columns with $T_{P}=100\;\mathrm{ns}$ width stimulation pulses at a rate of one pulse every 360 ns, we obtain an average current consumption of 49.52 μA at 3.3 V power supply. This corresponds to 16 (LRS) *s*ynaptic *op*erations (SOP) every 360 ns. Thus, the overall effective *e*nergy per (LRS) *s*ynaptic *op*eration $E_{\mathrm{SOP}}$ is given by^1^

8.1 $$E_{\mathrm{SOP}}=\frac{1}{16}\times 49.52\;\text{μ}A\times 3.3\;V\times 360\;\mathrm{ns}=3.7\;\mathrm{pJ}.$$

The breakdown of the 49.52 μA average current consumption is as follows: 29.49 μA (59.6%) is consumed by the memristors (1.84 μA by each of the 16 LRS memristors), 17.6 μA (35.58%) by the differential amplifiers driving them, 2.24 μA (4.5%) by the attenuator circuit, and 170 nA (0.3%) by the neurons. Note that the energy dissipated by an individual LRS memristor generating a $\delta q_{\mathrm{memr}}=0.68\;\mathrm{pC}$ charge packet is $E_{\mathrm{LRS}}=3.3\;V\times 0.68\;\mathrm{pC}=2.24\;\mathrm{pJ}$, about 60% of the energy in equation (8.1). The other 40% are contributed by the corresponding share of the rest of the circuitry. For scaled-up systems, in which the common circuitry is shared by more memristors, the resulting $E_{\mathrm{SOP}}$ value should slowly approach the baseline of $E_{\mathrm{LRS}}=2.24\;\mathrm{pJ}$ (or even less if LRS memristors are sparse). However, if scaling up aggressively, the combined differential amplifier in figure 2 and transistor $M_{h}$ may need to be redesigned for properly handling larger currents.

**Figure 18.**
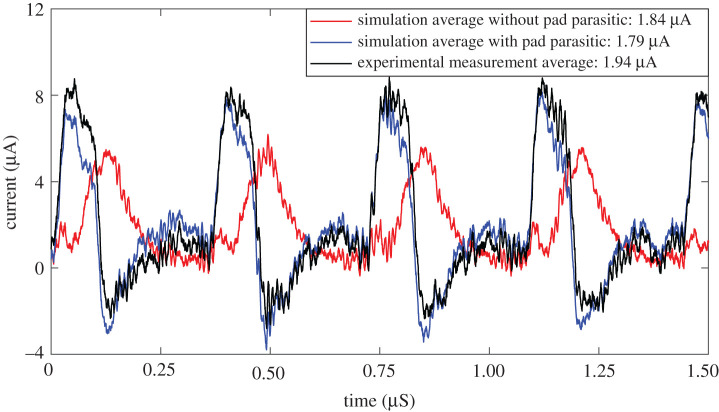
Currents flowing through one single LRS memristor, stimulated with 100 ns pulses at a rate of one pulse every 360 ns. (Online version in colour.)

The energy figures mentioned above were obtained by simulations, as we could not measure experimentally the detailed breakdown of the current consumption of all sub-circuits. However, the current consumption that we could measure experimentally was below 10% difference with respect to the simulated one. Additionally, our set-up allowed us to measure precisely the average current consumed by one single LRS memristor because both of its terminals were accessible in our hybrid multi-chip architecture. Figure 18 shows three current traces flowing through an LRS memristor. The black trace corresponds to an experimental measurement, yielding an average memristor current of 1.94 μA. The red trace corresponds to the simulation mentioned above, with an average of 1.84 μA. The black and red traces are quite different, although the average is very similar (5% difference). This is because in the experimental set-up, the top plate of the memristors is connected to two chip pads, PCB traces, and the oscilloscope probe, thus adding a large parasitic capacitance. This makes this node to move much slower than the bottom plate making the polarity of the memristor change sign, as well as the current, as can be seen in the black trace in figure 18. By adding in the simulation an extra 8 pF capacitor to the top plate node, the blue trace in figure 18 is obtained, which is almost identical to the experimental one, having an average of 1.79 μA. Therefore, a full monolithic realization would follow the red trace. However, the presence of the top plate very large parasitic capacitance in the experimental set-up is not affecting dramatically the average current, nor the average power consumption.^2^

For a fully integrated on-chip system, additional communication circuitry would be required to send spikes in and out, or to communicate spikes between on-chip computing crossbars. The energy consumption of such communication circuits is not considered in equation (8.1). Table 5 shows a comparison with some neural processing chips, spiking and non-spiking, using RRAM or SRAM for weight storage, that have been reported recently.

## 9. Conclusion

In this paper, we have shown the successful experimental operation of a small SNN that used a 4×4 1T1R memristor crossbar as synapses together with CMOS analogue neurons. We also used one compact MOS ladder-based current-splitter circuit per neuron to aggressively downscale the memristor-domain micro-amp current levels to the required analogue CMOS neuron-domain current levels. The fully experimental SNN was assembled using three separate chips. The first chip provided individual novel Ti/C/Au top-plate memristors with low set/reset voltage while presenting high OFF resistance. The second chip was fabricated in a standard 180 nm CMOS technology and provided the NMOS selector transistors required for all 1T1R synapses. The third chip, fabricated in the same 180 nm CMOS technology, provided the post-synaptic circuitry, including the current attenuator circuits and the neuron circuits. These three chips interacted with a custom PCB and an FPGA-PCB. The custom PCB provided all the analogue biases, which were independently adjustable, and the pre-synaptic stimulation pulses, while the FPGA-PCB digitally controlled all the switches and multiplexers on the custom PCB. The system was used to showcase two learning scenarios. One was based on one-shot WTA training, while the other implemented SB-STDP. Successful operation was demonstrated in both scenarios. The set-up is clearly very useful in that it facilitates experimentation with new custom-made memristors. Energy measurements reveal this approach as highly promising for ultra-low power systems. Although the hardware example cases shown are small-size from the computational point of view, they are capable of performing computations, such as SB-STDP, which have been demonstrated previously capable of solving much larger scale computing systems [13]. In order to substantially boost the computing capability and size of the current spiking neural network, further effort is required to fabricate multiple bit memristor devices monolithically on top of CMOS neurons [2].

## Data Availability

This article has no additional data.

## Footnotes

1 The $E_{\mathrm{SOP}}$ figure of merit is the inverse of another popular figure of merit used many times in neural processing systems, which is the ‘number of synaptic operations per second and per watt’.

2 This is so because the top plate node is a virtual ground node. In this case, a capacitor connected to it will always return whatever charge it absorbs temporarily, and therefore should not alter the total charge packets travelling through this node.
